# Inter-method reliability for determining total and soluble fluorides in child low-fluoride formula dentifrices

**DOI:** 10.1038/s41598-020-77920-3

**Published:** 2020-11-30

**Authors:** Mohammed Nadeem Bijle, James Tsoi, Manikandan Ekambaram, Edward C. M. Lo, Clifton M. Carey, Cynthia Kar Yung Yiu

**Affiliations:** 1 2/F, Paediatric Dentistry, Prince Philip Dental Hospital, Faculty of Dentistry, The University of Hong Kong, 34 Hospital Road, Hong Kong, Hong Kong SAR; 2 Dental Materials Science, Faculty of Dentistry, The University of Hong Kong, Hong Kong, Hong Kong SAR; 3 Paediatric Dentistry, Faculty of Dentistry, University of Otago, Dunedin, New Zealand; 4 Dental Public Health, Faculty of Dentistry, The University of Hong Kong, Hong Kong, Hong Kong SAR; 5 Craniofacial Biology, School of Dental Medicine, University of Colorado, Denver, CO USA

**Keywords:** Biochemistry, Chemistry

## Abstract

The study aimed to compare three methods for determining total (TF) and total soluble fluorides (TSF) in 5 child formula dentifrices (CFD) using Inter-method reliability (IMR) statistical approach. The methods were direct acid-hydrolysis (DM), the least-time-consuming method; Modified direct acid-hydrolysis with standard-addition method (MDM), ISO 19448:2018 method; and modified Taves acid-HMDS diffusion analysis (TAD), the claimed gold standard method. A significant difference in the mean difference was observed for all methods at all levels (*p* < 0.001), except DM and TAD for TF (*p* = 0.622). A proportional bias was discerned in the agreement distribution between DM and TAD for TF (*p* < 0.001)**.** The ICC analysis identified significant reliability between all measurements, irrespective of the model, measure, and fluoride type (*p* < 0.001). For TF and TSF, the IMR between DM and TAD was lower than MDM and TAD for consistency/absolute agreement reliability at single/average measures. The reliability measure for DM and MDM was higher than MDM and TAD for TSF, but was lower than MDM and TAD for TF. The ICC measure for DM-TAD was significantly lower than DM-MDM and MDM-TAD (*p* < 0.05). The ISO 19448:2018 MDM is a reliable test that can be used as an alternative to TAD/DM for determining TF/TSF in CFD.

## Introduction

Dental caries is a dynamic process caused by an imbalance between the net mineral loss (demineralization) and mineral gain (remineralization) of hard tissues of the teeth. Although remineralization is a natural process, fluorides (F) are known to inhibit demineralization by formation of fluorapatite and subsequently enhance the remineralization process^1^. There is general agreement that the availability of low levels of F in the oral cavity by continuous use of fluoridated toothpastes aid to inhibit demineralization and prevent the development of dental caries^2,3^. Despite the evident beneficial effect of fluoride-containing dentifrices^3^, around 486 million children worldwide suffer from dental caries of primary teeth^4^.

Irrespective of the variation in concentration of F in dentifrices, an estimate of total F (TF) and total soluble F (TSF) identifies the proportion of insoluble F content in the dentifrices, which is the fraction of unavailable or chemically insoluble F that restricts F bioavailability. A study on child formula dentifrices in Chile found considerably lower TSF as compared to TF in several low-F containing dentifrices, illustrating the presence of insoluble F, whereas those high-F dentifrices with mean F concentration > 1000 ppm had TF similar to the measured TSF^5^. Another study in Brazil showed that approximately 50% of the fluoride-containing dentifrices used by children contained insoluble or inactive fluorides^6^. The evidence suggests that dentifrices containing > 1000 ppm F are more effective in reducing dental caries as compared to non-fluoridated and low-fluoride (< 500 ppm) dentifrices. A recent Cochrane review reported an evident dose–response effect of fluoride dentifrices for caries prevention in children and adolescents^7^. However, considering the results of the previous studies^5,6^ it is possible that the low-fluoride child dentifrices (400–1000 ppm F) contain significantly less TF and TSF, different from the claims of the manufacturers, which might have further affected its caries-preventive potential.

The F compounds in most of the child formula F-containing dentifrices are sodium fluoride (NaF), sodium monofluorophosphate (NaMFP), and amine fluoride (amine F, commercially known as Olaflur, i.e. 2,2′-((3-((2-Hydroxyethyl)octadecylamino)propyl)imino)diethanol dihydrofluoride). Along with F compounds, the dentifrices also contain abrasives like silica, dicalcium phosphate dihydrate, calcium carbonate, aluminium silicate, and sodium silicate. The higher concentration of inactive or insoluble F is due to the reactive incompatibility between the F source and abrasive salts, hindering the bioavailability of F and toothpaste efficacy^8^. To discern formulations with limited F bioavailability, a reliable analytical method that can rapidly detect the concentration of insoluble F is needed.

Several regulatory agencies have specified the use of the F measurement method based on the Taves assay for estimating F concentration in dentifrices. The assay separates F from the complex samples by rapid diffusion and analyzes F using fluoride-ion selective electrode (F-ISE)^9^. Although the modified Taves diffusion analysis (TAD) has been considered as the gold standard for fluoride analysis, the procedure is time consuming and requires specialized apparatus (diffusion dishes). Other analytical methods suggested for estimating F concentrations in the dentifrices include direct acid-hydrolysis (DM), acid digestion-gas chromatography, and acid digestion–diffusion method; of which DM is the least time consuming and only requires basic laboratory equipments^10^. However, the method is not indicated for all F sources in the dentifrices, especially those with some profluoride compounds.

Recently, ISO Standard ISO 19448:2018 (Section 8.2) proposes that the standard addition technique is suitable for estimating F concentration in samples with bound F ion or when the unknown sample is contaminated with other substances that alter the assay. The DM method can easily be supplemented with the technique to overcome its limitations as it just requires some additional steps for sample preparation and calculations (Section 10.2 of ISO 19448:2018). The method can be addressed as the modified direct acid-hydrolysis with standard-addition technique (MDM). However, no study have compared the reliability of MDM to DM or the gold standard TAD for fluoride analysis .

Thus, the objective of this study was to statistically assess the Inter-method reliability (IMR) of three methods, i.e. DM, MDM, and TAD for determining TF and TSF in child formula dentifrices. The null hypothesis tested in the present study was that there is no difference in the IMR among DM, MDM, and TAD; thus, all methods are equally reliable to determine total and total soluble fluorides in low-fluoride-containing child formula dentifrices.

## Results

### Total and total soluble fluorides

**Figure 1:**
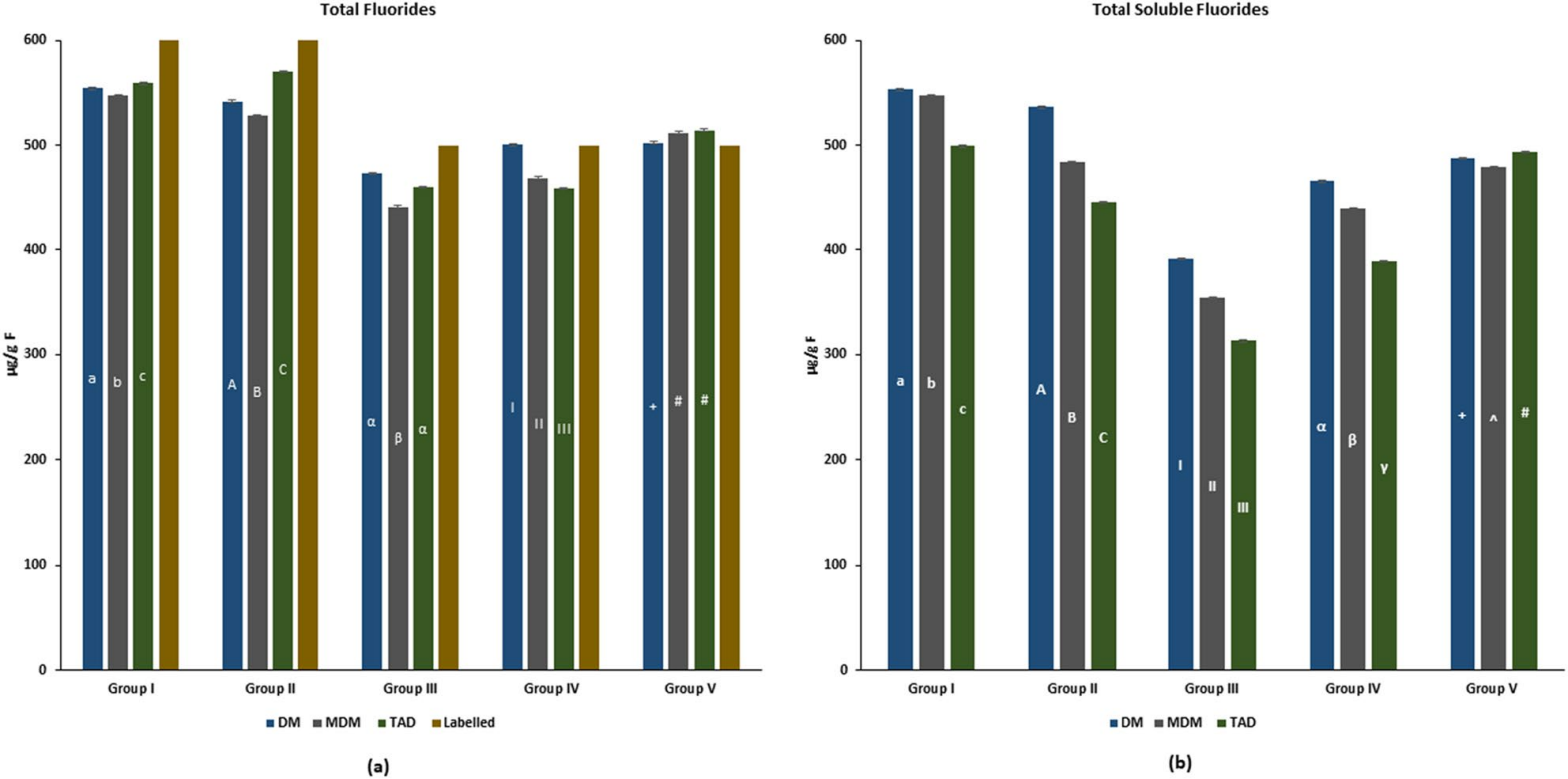
Total and total soluble fluorides estimated using direct acid-hydrolysis method, modified direct acid-hydrolysis method, and Taves acid diffusion analysis (n = 3 per group/experiment). (Small alphabets, capital alphabets, Greek alphabets, Roman numerals, and signs indicate significant differences between the methods for estimating fluorides).

The data for the estimated TF and TSF in the child dentifrices with different methods is presented in Fig. 1a,b, respectively.

Except for Group V (where all methods exceeded the labelled F concentrations), the F concentrations estimated by all three methods were lower than the labelled F concentrations. For Group IV, the F concentrations estimated by DM was similar to the labelled F concentrations.

The TF of Group I and Group II (600 ppm F-containing) dentifrices, estimated by TAD was significantly higher than the other methods (*p* < 0.05). Whereas, for Group IV, the TF estimated with TAD was significantly lower than the other methods (*p* < 0.05). In Group III, the TF estimated by TAD and DM was significantly higher than MDM (*p* < 0.05); while for Group V, the TF assessed by TAD and MDM was significantly higher than DM (*p* < 0.05).

The TSF estimated by TAD for all the groups (except Group V) was significantly lower than the other methods (*p* < 0.05). Conversely, the TSF analysed using DM (except Group V) was significantly higher than the other methods (*p* < 0.05). For Group V, the TSF by TAD was significantly higher than the DM > MDM (*p* < 0.05).

Irrespective of the fluoride type (TF/TSF), the F concentration estimated by all the methods in majority of groups was significantly different from each other, showing inconsistency in estimation of similar/identical F concentrations.

### Inter-method agreement

**Figure 2:**
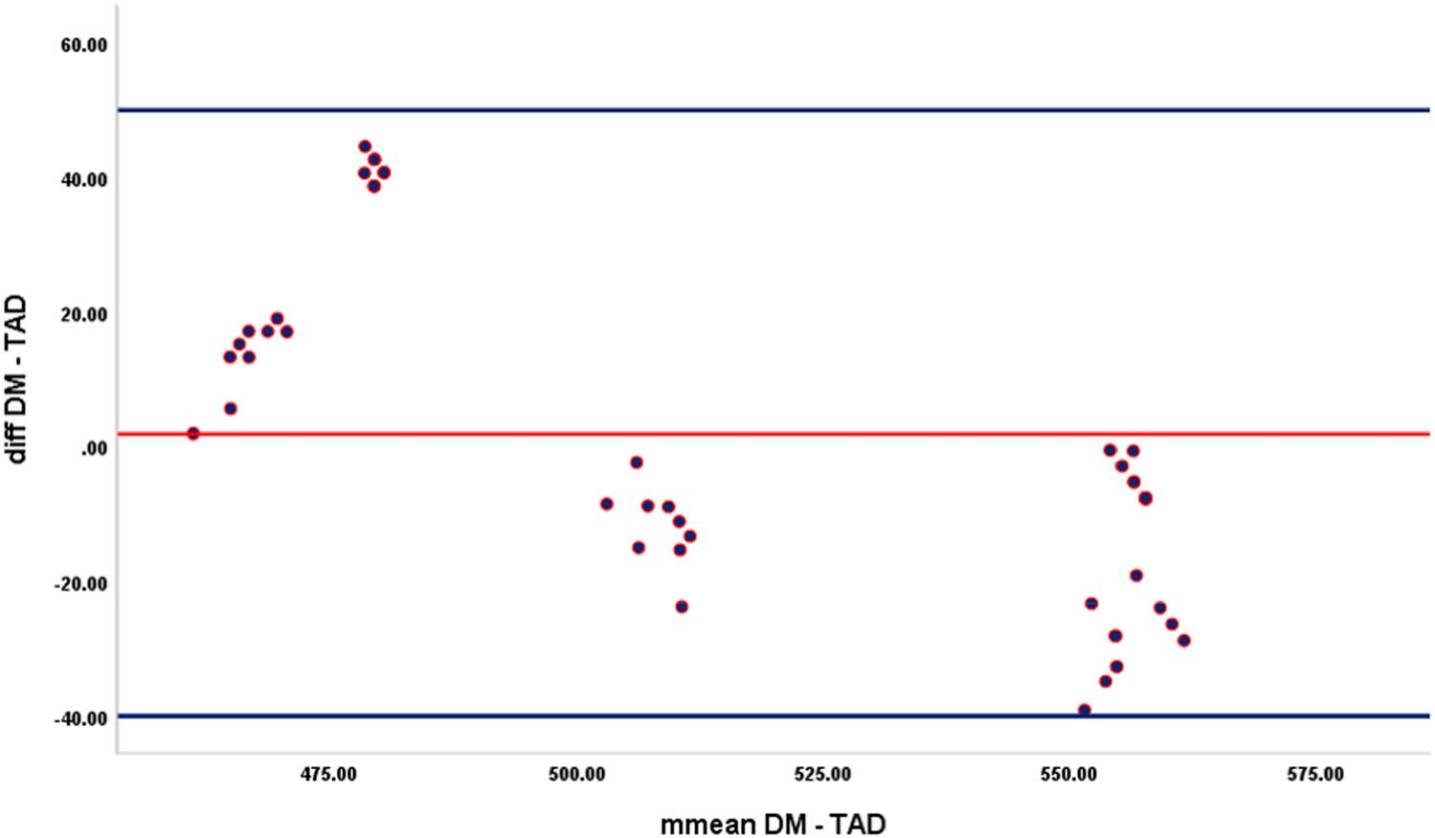
Bland–Altman plot for agreement distribution between direct acid-hydrolysis method and Taves acid diffusion analysis for estimated total fluorides.

The data for inter-method agreement as per Bland–Altman analysis is presented in Table 1. A significant difference in the mean difference among methods was observed for all methods at all levels, including the combined TF and TSF (*p* < 0.001), except between DM and TAD for TF (*p* = 0.622). A proportional bias was discerned in the agreement distribution between DM and TAD for TF (*p* < 0.001), irrespective of log transformation and re-assessment of the agreement distribution with linear regression analysis between the mean difference and inter-measurement mean in the model (Fig. 2).

**Table 1:** Inter-method agreement between direct acid-hydrolysis method, modified direct acid-hydrolysis method, and Taves acid diffusion analysis for estimated total and total soluble fluorides using Bland–Altman analysis.

Groups	Mean difference (µg/g F)	95% CI—LL	95% CI—UL	*p *value
**Total fluorides**				
DM-MDM	15.04	9.12	20.96	< 0.001
DM-TAD	1.82	− 5.56	9.19	0.622
MDM-TAD	− 13.22	− 19.59	− 6.86	< 0.001
**Total soluble fluorides**				
DM-MDM	25.92	20.46	31.38	< 0.001
DM-TAD	58.60	48.06	69.15	< 0.001
MDM-TAD	32.68	25.33	40.04	< 0.001
**Combined total and total soluble fluorides**				
DM-MDM	20.48	16.37	24.59	< 0.001
DM-TAD	30.21	21.52	38.90	< 0.001
MDM-TAD	9.73	2.94	16.52	< 0.001

Data was analysed using 1-sample t-test; *p* < 0.05 is significant. *CI* confidence interval, *LL* lower limit,* UL* upper limit.

The results of Bland–Altman analysis demonstrated that irrespective of the F types, all the methods did not achieve identical F concentration measurements, which is in concordance with the results of 1-way ANOVA.

### Inter-method reliability (IMR)

The inter-method reliability analysis using ICC reliability index by assessing single or average measures for TF, TSF, and combined TF and TSF is presented in Tables 2, 3 and 4, respectively. Table 5 shows the statistical analysis for between-method ICC measures performed by Feldt’s test.

**Table 2:** Inter-method reliability using intra-class correlation coefficient reliability index between direct acid-hydrolysis method, modified direct acid-hydrolysis method, and Taves acid diffusion analysis for estimated total fluorides.

Total fluorides				
Groups	ICC	95% CI—LL	95% CI—UL	*p* value
**Single measures**				
Measurement type: consistency				
DM–TAD	0.81	0.68	0.89	< 0.001
MDM–TAD	0.89	0.80	0.94	< 0.001
DM–MDM	0.85	0.74	0.92	< 0.001
Measurement type: absolute agreement				
DM–TAD	0.82	0.69	0.89	< 0.001
MDM–TAD	0.85	0.63	0.93	< 0.001
DM–MDM	0.78	0.41	0.91	< 0.001
**Average measures**				
Measurement type: consistency				
DM–TAD	0.90	0.81	0.94	< 0.001
MDM–TAD	0.94	0.89	0.97	< 0.001
DM–MDM	0.92	0.85	0.96	< 0.001
Measurement type: absolute agreement				
DM–TAD	0.90	0.81	0.94	< 0.001
MDM–TAD	0.92	0.78	0.96	< 0.001
DM–MDM	0.88	0.58	0.95	< 0.001

*ICC* intra-class correlation coefficient, *CI* confidence interval, *LL* lower limit, *UL* upper limit; *p* < 0.05 is significant.

ICC measures interpretation: 0.00 to 0.20: slight; 0.21 to 0.40: fair; 0.41 to 0.60: moderate; 0.61 to 0.80: substantial; and 0.81 to 1.00: almost perfect correlation.

**Table 3:** Inter-method reliability using intra-class correlation coefficient reliability index between direct acid-hydrolysis method, modified direct acid-hydrolysis method, and Taves acid diffusion analysis for estimated total soluble fluorides.

Total soluble fluorides				
Group	ICC	95% CI—LL	95% CI—UL	*p* value
**Single measures**				
Measurement type: consistency				
DM–TAD	0.85	0.75	0.92	< 0.001
MDM–TAD	0.93	0.88	0.96	< 0.001
DM–MDM	0.96	0.92	0.98	< 0.001
Measurement type: absolute agreement				
DM–TAD	0.61	− 0.09	0.86	< 0.001
MDM–TAD	0.84	0.08	0.95	< 0.001
DM–MDM	0.88	0.10	0.97	< 0.001
**Average measures**				
Measurement type: consistency				
DM–TAD	0.92	0.85	0.96	< 0.001
MDM–TAD	0.97	0.94	0.98	< 0.001
DM–MDM	0.98	0.96	0.99	< 0.001
Measurement type: absolute agreement				
DM–TAD	0.75	− 0.19	0.93	< 0.001
MDM–TAD	0.91	0.14	0.97	< 0.001
DM–MDM	0.94	0.18	0.98	< 0.001

*ICC* intra-class correlation coefficient, *CI* confidence interval, *LL* lower limit, *UL* upper limit; *p* < 0.05 is significant.

ICC measures interpretation: 0.00 to 0.20: slight; 0.21 to 0.40: fair; 0.41 to 0.60: moderate; 0.61 to 0.80: substantial; and 0.81 to 1.00: almost perfect correlation.

**Table 4:** Inter-method reliability using intra-class correlation coefficient reliability index between direct acid-hydrolysis method, modified direct acid-hydrolysis method, and Taves acid diffusion analysis for estimated total and total soluble fluorides.

Combined total and total soluble fluorides				
Group	ICC	95% CI—LL	95% CI—UL	*p* value
**Single measures**				
Measurement type: consistency				
DM–TAD	0.78	0.68	0.85	< 0.001
MDM–TAD	0.88	0.82	0.92	< 0.001
DM-MDM	0.93	0.90	0.95	< 0.001
Measurement type: absolute agreement				
DM–TAD	0.70	0.34	0.84	< 0.001
MDM–TAD	0.87	0.80	0.92	< 0.001
DM-MDM	0.87	0.34	0.95	< 0.001
**Average measures**				
Measurement type: consistency				
DM–TAD	0.87	0.81	0.92	< 0.001
MDM–TAD	0.94	0.90	0.96	< 0.001
DM-MDM	0.96	0.95	0.98	< 0.001
Measurement type: absolute agreement				
DM–TAD	0.82	0.50	0.92	< 0.001
MDM–TAD	0.93	0.89	0.96	< 0.001
DM–MDM	0.93	0.51	0.98	< 0.001

*ICC* intra-class correlation coefficient, *CI* confidence interval, *LL* lower limit, *UL* upper limit; *p* < 0.05 is significant.

ICC measures interpretation: 0.00 to 0.20: slight; 0.21 to 0.40: fair; 0.41 to 0.60: moderate; 0.61 to 0.80: substantial; and 0.81 to 1.00: almost perfect correlation.

**Table 5:** Analysis of derived reliability measures.

Groups	*p* value			
	Single measures		Average measures	
	Consistency	Absolute agreement	Consistency	Absolute agreement
**Total fluorides**				
DM–TAD	0.344	0.677	0.307	0.650
MDM–TAD				
DM–MDM	0.597	0.481	0.575	0.441
MDM–TAD				
DM–TAD	0.673	0.772	0.641	0.750
DM–MDM				
**Total soluble fluorides**				
DM–TAD	0.137	0.104	0.115	0.063
MDM–TAD				
DM–MDM	0.450	0.589	0.466	0.558
MDM–TAD				
DM–TAD	0.028*	0.033*	0.024*	0.017*
DM–MDM				
**Combined total and total soluble fluorides**				
DM–TAD	0.105	0.024*	0.078	0.013*
MDM–TAD				
DM–MDM	0.128	0.935	0.115	0.940
MDM–TAD				
DM–TAD	0.002*	0.029*	0.001*	0.016*
DM–MDM				

Reliability measures were analysed using Feldt's test; *p* < 0.05 is significant indicated by*.

Irrespective of the assessed measures and measurement type for TF, the reliability coefficient between MDM and TAD (single measure ≥ 0.85; average measures ≥ 0.92) was higher than the other between methods reliability measure (Table 2). For consistency measurements analysis, the ICC between DM and TAD was the smallest; whereas for absolute agreement, the reliability measure for DM and MDM was the lowest.

For TSF, the ICC reliability index between DM and MDM (single/average measures ≥ 0.88) was the highest, followed by MDM and TAD and the lowest measures were estimated between DM and TAD for all measurement types assessed for single or average measures (Table 3). For absolute agreement, the coefficient measures between DM and TAD was < 0.80, but ≥ 0.61, indicating a shift in the reliability index from almost perfect to substantial reliability measures (Table 3).

The inter-method reliability for combined TF and TSF in the consistency measurement was the highest between DM and MDM, followed by between MDM and TAD, with the least coefficient measures between DM and TAD (Table 4). Similarly, for absolute agreement measurements, the reliability scores between DM and TAD were the least, whereas, identical reliability measures were observed for between DM–MDM and MDM–TAD.

As per Feldt’s test, a significant difference in the reliability measures was identified between DM–TAD and DM–MDM for all measures and measurement types for analysis of TSF; of which DM–MDM coefficients were significantly higher than the DM–TAD coefficients (*p* < 0.05). Similarly, the ICC for DM–MDM was significantly higher than DM–TAD for combined TF and TSF, irrespective of the measures and measurement types (*p* < 0.05). Further, for combined TF and TSF, the absolute agreement measurement irrespective of the assessed measure, the reliability measures for MDM–TAD was significantly higher than those for the DM–TAD (*p* < 0.05) (Table 5).

Thus, the overall inter-method reliability results showed that the MDM demonstrated higher reliability to the gold standard TAD as compared to the DM for estimated TF and TSF.

### Effect of factor interaction on estimated fluorides

Three-way ANOVA test revealed that the group, method, fluoride type, the interaction between group-method, group-fluoride type, method-fluoride type, and group-method-fluoride type had a significant effect on the estimated F concentrations (*p* < 0.001). Table 6 shows the effect of group interaction with methods on the estimates of F concentrations. The F concentrations for DM was significantly higher for Group I than the other groups, followed by Group II > Group IV = Group V > Group III (*p* < 0.05). However, the estimates of F concentration by both MDM and TAD for Group I was significantly higher than other groups followed by Group II = Group V > Group IV > Group III (*p* < 0.05).

**Table 6:** Effect of the child formula dentifrices and methods on the estimated fluoride concentrations.

Methods	Mean estimates in µg/g F (95% confidence interval)					Multiple comparison
	Group I	Group II	Group III	Group IV	Group V	
DM	553.63 (547.37–559.89)	539.10 (532.84–545.36)	432.42 (426.16–438.68)	482.85 (476.59–489.11)	494.84 (488.58–501.10)	Group I > Group II > Group IV = Group V > Group III
MDM	547.18 (540.92–553.44)	505.97 (499.71–512.23)	397.82 (391.55–404.07)	454.12 (447.86–460.38)	495.35 (489.09–501.61)	Group I > Group II = Group V > Group IV > Group III
TAD	529.19 (522.93–535.45)	508.00 (501.74–514.26)	386.82 (380.40–392.92)	424.01 (417.75–430.27)	503.92 (497.66–510.18)	Group I > Group II = Group V > Group IV > Group III

Three-way ANOVA with multiple comparisons by Bonferroni's post-hoc test for ANOVA; *p* < 0.05 is significant.

The interaction effect on F concentrations revealed that TAD and MDM were able to discern similar differences between different child dentifrices; whereas DM was unique to the other methods.

All inclusive, the results of the study suggest that MDM has the potential to identify TF and TSF similar to that of TAD; whereas reliability of DM to the gold standard TAD is lower than that of MDM.

## Discussion

In the present study, the F concentration estimates for TF and TSF in the commercially-available low-fluoride child formula dentifrices were analysed using different methods, i.e. DM, MDM, and TAD. The comparative assessment for reliability measures demonstrated that MDM had higher reliability to the gold standard TAD than DM. Hence, the null hypothesis tested in the present study that all three methods (DM, MDM, and TAD), irrespective of F types, are capable of identifying identical and consistent F concentrations was rejected. The study results delineate that MDM could be an alternative to TAD for estimating TF and TSF, which requires less time, reagents, and apparatus than TAD.

In the present study, it is noteworthy that none of the methods (except for Group V) estimated TF concentrations similar or equal to the labelled F concentrations. A possible reason for the methods to have higher estimated TF concentrations in Group V is the dentifrice being a simple NaF toothpaste has few interferences as opposed to other child formula dentifrices investigated in the study. Only the TF estimated by DM in Group IV was similar to the labelled F concentration; while all the other methods showed lower F concentrations. Similarly, a potential reason for such results could be attributed to the inactive ingredients in the toothpaste matrix. However, in case of Group IV, the matrix constituents are unknown, and thus, the results remain unexplained. Another possible reason for the difference between TF estimated and labelled could be the manufacturing process of the dentifrices that has altered the TF concentrations.

This study aimed to assess the IMR between three methods. It is noteworthy that IMR is a statistical approach to estimate and indicate the degree of measurement error associated with the methods and not the validity^11^. A study used correlation estimates between expected and detected F concentrations with DM to show the method validity^12^. However, in this study the gold standard method (TAD) measures were used for the purpose of comparative assessment, that had no effect on the reliability measures analysis. The purpose of using the TAD was simply to analyse how closely related or consistent, other methods determine F concentration in the child formula dentifrices with respective to TAD. Thus, the reliability and agreement coefficients reported are relevant to the experimental objectives of the study and do not indicate method validity reference to the gold standard.

In the present study, it was observed that there was no between methods agreement to estimate TF and TSF in the child formula dentifrices, even though the F-ISE calibration stability was monitored. First, identical F concentrations between methods appears impractical as the included variables required by each method to prepare samples are unique. Second, the internal standards prepared per experiment will append further variability in estimating the F concentration as the calibration curve is subjected to variation. The TAD method is known to exhibit sufficient reproducibility with a standard deviation of 5%^10^. The DM has demonstrated low variability and high validity with the variation of approximately 1% for determining TF and TSF^5,6,12,13^. Therefore, the evident intra-method variability could be an additive factor for non-identical determination of TF and TSF in this study. However, expecting identical measures from different methods with different samples at separate instance appears unfeasible and unrealistic. Thus, the concern on the results of inter-method agreement appears non-problematic.

While analyzing agreement, the mean difference for TF estimates between DM and TAD was non-significant, indicating potential agreement between the methods. However, the linear regression analysis delineated a proportional bias in the mean difference. On careful observation, it was identified that the mean difference between groups proportionally favored integer balance across the observations and thus, the bias could be observed, which retained even following data log transformation. Hence, it was distinctly certain that no methods had shown potential agreements which is uncritical to the determined F concentrations by the methods.

The inter-method reliability results showed that the ICC measure between MDM and TAD was consistently higher than DM and TAD, irrespective of the estimated F concentration (i.e. TF or TSF or combined TF and TSF), measure, and measurement type for the adopted ICC model and analysis. For TSF and the combined TF and TSF, the reliability measures for DM–TAD were < 0.80 at certain reliability output measures and measurement types, indicating substantial reliability as opposed to the other measures, which shows almost perfect correlation between methods (> 0.80). One of the dentifrices included in the study contained amine F, whereby F source incompatibility with the method might have contributed to lower reliability measures of DM with TAD. The reliability measures for DM and MDM computed for TSF and the combined TF and TSF were higher than other between methods reliability index, demonstrating a close correlation between the methods as MDM is merely a modification of DM. However, this was not observed for the estimated TF that can be attributed to the sample preparation process and the claim for use of standard addition technique. The TSF sample preparation process involved additional steps of centrifugation; whereby the excipients were propelled to the sediments and the supernatant contained the active ingredients. As aforementioned, the standard addition technique is suitable for bound F ion or samples with additional substances (e.g. dentifrices) that influenced the detection of TF by MDM as opposed to DM.

Considering the definition of inter-method reliability highlighted in the present study, the results of factor interaction on estimated F concentration showed that MDM and TAD were similarly able to differentiate between different low-fluoride containing dentifrices based on the estimated F concentrations. The analysis further conforms to the results of the derived reliability index, which were analysed by Feldt’s test. The Feldt’s test is a statistical comparison method for reliability coefficients that aims to identify differences between derived measures. The between method reliability measures for F concentration estimates, irrespective of TF/TSF, for MDM–TAD was significantly higher than DM–TAD, which further reaffirmed that the MDM demonstrated higher reliability to TAD than DM. It is also noteworthy that for MDM–TAD the reliability index for average measures was > 0.90 which crosses the minimum to consider the correlation/agreement almost perfect for reliability as agreed by previous studies^11,14,15^. Hence, the results of the present study at multiple outcomes indicated that the reliability of MDM to TAD was significantly higher than DM for determination of TF and TSF in the child dentifrices.

Rapid estimation of TF and TSF with MDM will aid reliable detection of low concentration F in the child formula dentifrices. In addition, the method is independent of the assumption by TAD that the diffusion efficiency of samples and internal standards are the same as the sample diffusion efficiency of ~ 80% in TAD^10^. Apart, the addition of standards to the samples (in MDM) makes the concentration reach the best determination range for analyses with F electrode*.* Both the methods (TAD and MDM) require additional equipment/instruments/chemicals for the analysis; while, they are needed as the use of DM is limited in toothpastes with unknown matrix or profluoride compounds. Although internal standards preparation is needed in the MDM, the standards are not required to undergo overnight diffusion with perchloric acid as needed in TAD. However, the inter- and intra-laboratory reproducibility of the method needs to be addressed as no literature could be retrieved on MDM that provides this information. Hence, further cross-laboratory investigations are needed to provide more insight on the reproducibility and validity of MDM for estimation of TF and TSF, irrespective of the F concentration.

## Methods

### Reliability and agreement

The manuscript was prepared conforming to the Guidelines for Reporting Reliability and Agreement Studies (GRRAS)^11^. The GRRAS checklist highlighting reported items in the present study and manuscript is presented in Supplementary Table S1.

Inter-method agreement was defined as the degree to which the methods are capable of measuring identical F concentrations in different child formula dentifrices, respective/irrespective to the F types.

Inter-method reliability was defined as the ability of the methods to differentiate between different child formula dentifrices based on the estimated F concentrations, respective/irrespective to the F types.

### Fluoride determination

The F concentrations were estimated using F-ISE (Thermo Fisher Scientific, MA, USA) attached to a benchtop potentiometer (Orion 2700, Oakton Instruments, IL, USA) that was subjected to calibration with respective internal standards as per the method of determining F concentration in the dentifrices. For all experiments, the internal standards were prepared using 0.1, 1, 10, 100, and 1000 µg/g F per analysis. A linear regression between known F concentrations in the standards and determined mV was performed to estimate the F concentration in the tested dentifrices (R^2^ > 0.99). The F-ISE calibration stability was monitored by analysis of standards before, during and after the sample measurements. While estimating F concentrations, the samples/standards were stirred at 250 rpm on a magnetic stirrer using micro-magnetic bars. After each measurement, the F-ISE was thoroughly rinsed in deionized water (DIW) and gently blot dried using a dry fibreless napkin (Kimwipes Ex-L, Kimberley-Clark Professional, USA) to prepare the electrode for the next measurement. Except for the test solutions, precaution was taken to avoid contact with the electrode membrane in order to prevent any damage.

### Child formula dentifrices

Five representative commercially-available child formula dentifrices (Supplementary Table S2) were purchased with the claimed fluorides from the local supermarket and used in the study:

- Group I—Colgate kids anticavity toothpaste, Minions (600 ppm F—NaF).
- Group II—Darlie Jolly Junior (600 ppm F—NaMFP).
- Group III—Elmex Kinder Zahnpasta (500 ppm F—Amine F).
- Group IV—Lion Kodomo (500 ppm F—NaF with 5% Xylitol).
- Group V—Oral-B kids toothpaste (500 ppm F—NaF).

### Total and total soluble fluorides

For the present study, TF and TSF were defined based on a recently published workshop paper on methodology for determining potentially available fluorides in dentifrices^10^:

Total fluorides (TF) was defined as the total amount of fluorides present in the dentifrices measurable by the currently available methods.

Total soluble fluorides (TSF), also known as potentially available fluorides, was defined as the fraction of TF in the dentifrice formulation that is chemically soluble in water or acid.

### Direct acid-hydrolysis method (DM)

The DM used in the present study was as per a previously published paper^6^. In brief, the dentifrice slurries were prepared in 1:100 dilution in DIW. Prior to dilution, ~ 100 mg of the dentifrices were weighed in the centrifuge tube. The diluted dentifrices were thoroughly vortexed for 60 s to obtain a homogenized suspension. The suspension was used to determine TF in the respective dentifrices, whereas for TSF, the suspension was further centrifuged at 5000 × *g* for 10 min to receive the supernatant.

For TF, 0.25 mL of suspension was primarily acid hydrolyzed with 0.25 mL of 2.0 mol/L HCl for 1 h at 45 °C in an incubator. Then, the hydrolyzed suspension was neutralized with 0.5 mL of 1.0 mol/L NaOH and thoroughly mixed. The neutralized suspension was further buffered with 1 mL of total ionic strength adjustment buffer II (TISAB II) prior to analysis by F-ISE.

To estimate the TSF in the dentifrices, 0.25 mL of supernatant was acid hydrolyzed and neutralized with HCl (2 mol/L) and NaOH (1 mol/L), respectively (similar to the procedure described for TF sample preparation), prior to supplementing the neutralized supernatant with TISAB II (1 mL). The buffered supernatant was then subjected to F concentration estimation based on calibration curve prepared using internal standards.

The F-ISE calibration was performed using the freshly prepared internal standards with the same reagents as in the DM experimental protocol for preparation of TF and TSF samples.

### Modified direct acid-hydrolysis with standard-addition method (MDM)

The MDM is the modification of DM, which the samples for TF and TSF determination in the dentifrices were prepared as per the procedure for DM. The modification for DM (i.e. MDM) in the present study is based on the use of standard additions technique (ISO 19448:2018), in contrast to direct analysis technique in the DM.

A standard addition series of test samples and a sample with DIW in the ratio of 4:1 were prepared. The standards for the test samples addition were prepared from 1, 10, 100, and 1000 µg/g F standards. The samples were further buffered with equal volume of TISAB II prior to determining F concentration.

The calibration curve of standard solutions (as prepared for DM) were used to estimate F concentrations in the TF and TSF samples with the standard addition series. A plot was constructed of the added fluoride for each sample versus the measured F concentration for the samples based on the constructed calibration curve (R^2^ > 0.99). The F concentration in the sample was calculated from the plotted linear regression by dividing the y-intercept (b) by the slope (m) as per the regression equation: y = mx + b. Thus, the absolute value or modulus of the x-intercept is the F concentration in the test solution which was further adjusted for the respective dilution factor.

### Modified Taves acid-HMDS diffusion analysis (TAD)

The modified Taves^9^ acid-diffusion analysis used in the present study was addressed as the gold standard for determining the TF and TSF in the dentifrices. The dentifrice slurries prepared in the method simulated conditions for use of dentifrices in the oral environment.

The slurry was prepared in 1:3 ratio with DIW, whereby 10 g of toothpaste was diluted in 30 mL DIW. The suspension was thoroughly homogenized by vortexing for 60 s. To avoid pipetting errors with the foamy consistency of the slurries, the test solutions were carefully weighed on a single balanced scale prior to diffusion. For TF, dentifrice slurries were used to estimate the F concentration, while the slurries were further centrifuged at 5000 × *g* for 2 min to obtain the supernatant which was used to estimate TSF in the child dentifrices.

The method was performed on Conway Diffusion Dishes (Bel-Art, Thermo Fisher Scientific, Sweden) lubricated with petroleum jelly (Vaseline, Unilever, UK) in the outermost compartment which held the lid of the dishes to obtain thorough seal. The next adjacent compartment inwards was supplemented with 4 mL of 1.0 mol/L perchloric acid (HClO_4_) saturated with 2.5% hexamethyldisiloxane (HMDS) and the test solution. Caution was taken on the addition of the test solution until the dishes were prepared for seal to prevent leakage of HMDS-F complex. The innermost compartment was supplemented with 0.5 mL of 1.0 mol/L KOH. Before placing the lid, 0.5 mL of the test solution was added to the compartment containing HClO_4_ and the dishes were immediately ensured to be sealed. Then, the dishes were overnight (~ 18 h) tilt-mixed on an orbital shaker (Labnet, Woodbridge, USA) at 60 rpm at room temperature to permit adequate diffusion of the samples. After overnight diffusion, the F trapped KOH from the samples were neutralized with 1 mol/L HCl and thereafter buffered with 1 mL of TISAB II. The buffered solution was then subjected to F estimation through calibrated F-ISE. The standards prepared for the calibration underwent the same diffusion steps as those for test solutions preparation since the diffusion of F from the samples to KOH is not 100% efficient. The internal standards were then subjected to calibration curve to determine the sample F concentrations.

### Statistical analysis

All experiments were done in triplicate using different dentifrice tubes. The estimated fluoride concentrations in µg/g was entered in MS Excel 2016 worksheet (Office Professional Plus, Microsoft, USA) which was further subjected to statistical analysis using SPSS ver. 25 (IBM Statistics Inc., USA).

One-way ANOVA with Tukey’s HSD post-hoc test was used to analyse the effect of different methods on TF and TSF estimated in child dentifrices.

Between-methods agreement was quantified and described using Bland–Altman plot and further analysed by one-sample t-test with further proportional bias analysis by linear regression at *p* < 0.05. Following linear regression analysis to determine proportional bias, data was log transformed and re-analysed by one-sample t-test and linear regression analysis for inter-method agreements with identified proportional bias. The linear regression included mean difference as the dependent variable and between method measurement mean as the independent variable in the model.

Inter-method reliability was assessed using intra-class correlation coefficient statistics (ICC) for single and average measures using two-way mixed effects model for consistency and absolute agreement. The derived reliability measures were further analysed using Feldt’s test^16^ at α = 0.05.

Three-way ANOVA was used to analyse the effect of three factors (Factor 1—dentifrice, Factor 2—method, Factor 3—fluoride type) on the estimated fluoride concentrations primarily aimed to identify the effect of interaction between factors on the dependent variable (F concentration).

## Conclusion

Under the conditions of the experiments in this study, we conclude that the ISO 19448:2018 modified direct acid-hydrolysis with standard-addition method is reliable for determining total and total soluble fluorides in child formula low-fluoride dentifrices.

## Supplementary information

Supplementary Information.
