# Supplementary material for: Inter-method reliability for determining total and soluble fluorides in child low-fluoride formula dentifrices
Source: Sci Rep. 2020 Nov 30;10:20880. doi: 10.1038/s41598-020-77920-3 (PMC7705677; doi:10.1038/s41598-020-77920-3)
Supplement: Supplementary file 1 — Supplementary Information. [file 41598_2020_77920_MOESM1_ESM.docx]

**Title of the manuscript:** Inter-method reliability for determining total and soluble fluorides in child low fluoride formula dentifrices

**Name(s) of the author(s):**

**Mohammed Nadeem Bijle^1^, James Tsoi^2^, Manikandan Ekambaram^3^, Edward C M Lo^4^, Clifton M Carey^5^, Cynthia Kar Yung Yiu^6^**

**Affiliation(s) and address(es):**

^1^PhD Candidate, Paediatric Dentistry, Faculty of Dentistry, The University of Hong Kong, Hong Kong. [mnbijle@connect.hku.hk](mailto:mnbijle@connect.hku.hk) (ORCID: 0000-0002-7205-9891).

^2^Assistant Professor in Dental Materials, Faculty of Dentistry, The University of Hong Kong, Hong Kong. [jkhtsoi@hku.hk](mailto:jkhtsoi@hku.hk) (ORCID: 0000-0002-0698-7155)

^3^Senior Lecturer, Paediatric Dentistry, Faculty of Dentistry, University of Otago, Dunedin, New Zealand. [mani.ekambaram@otago.ac.nz](mailto:mani.ekambaram@otago.ac.nz) (ORCID: 0000-0002-9156-3727)

^4^Clinical Professor in Dental Public Health, Faculty of Dentistry, The University of Hong Kong, Hong Kong. [edward-lo@hku.hk](mailto:edward-lo@hku.hk) (ORCID: 0000-0002-2518-2686)

^5^Professor in Craniofacial Biology, School of Dental Medicine, University of Colorado, Colorado, United States of America. [Clifton.carey@cuanschutz.edu](mailto:Clifton.carey@cuanschutz.edu) (ORCID: 0000-0002-5871-0190)

^6^Clinical Professor in Paediatric Dentistry, Faculty of Dentistry, The University of Hong Kong, Hong Kong. [ckyyiu@hku.hk](mailto:ckyyiu@hku.hk) (ORCID: 0000-0003-4090-6205)

**Corresponding author:**

**Professor Cynthia Yiu.**

2/F, Paediatric Dentistry,

Faculty of Dentistry, The University of Hong Kong,

34 Hospital Road, Prince Philips Dental Hospital,

Sai Ying Pun, Hong Kong Island, Hong Kong.

Email: [ckyyiu@hku.hk](mailto:ckyyiu@hku.hk). Tel no: +852 2859 0256.

**Table S1: GRRAS Checklist**

| **Section** | **Item** | **GRRAS Checklist Item** | **Reported on Page No.** |
| --- | --- | --- | --- |
| **TITLE AND ABSTRACT** | | | |
| Title & Abstract | 1 | Identify in title or abstract that interrater/intrarater reliability or agreement was investigated. | 1,2,3 |
| **INTRODUCTION** | | | |
| Diagnostic/Measurement Device | 2 | Name and describe the diagnostic or measurement device of interest explicitly. | 4 |
| Population of interest | 3 | Specify the subject population of interest. | 3,4 |
| Rater population | 4 | Specify the rater population of interest (if applicable). | NA |
| Study rationale | 5 | Describe what is already known about reliability and agreement and provide a rationale for the study (if applicable). | NA |
| **METHODS** | | | |
| Sample size | 6 | Explain how the sample size was chosen. State the determined number of raters, subjects/objects, and replicate observations. | 8 |
| Sampling method | 7 | Describe the sampling method. | NA |
| Measurement process | 8 | Describe the measurement/rating process (e.g. time interval between repeated measurements, availability of clinical information, blinding). | 4-8 |
| Independent measurements (**Yes**) | 9 | State whether measurements/ratings were conducted independently. | 4-8 |
| Statistical analysis | 10 | Describe the statistical analysis. | 8 |
| **RESULTS** | | | |
| Observations | 11 | State the actual number of raters and subjects/objects which were included and the number of replicate observations which were conducted. | 8-10 |
| Rater/Subject characteristics | 12 | Describe the sample characteristics of raters and subjects (e.g. training, experience). | NA |
| Agreement/Reliability estimates | 13 | Report estimates of reliability and agreement including measures of statistical uncertainty. | 8-10 |
| **DISCUSSION** | | | |
| Relevance of results | 14 | Discuss the practical relevance of results. | 10-13 |
| **AUXILIARY MATERIAL** | | | |
| Detailed results | 15 | Provide detailed results if possible (e.g. online) | 16-23 |
| NA – Not Applicable | | | |

**Table S2: Child formula dentifrices used in the present study.**

| **Experimental Groups** | **Product** | **Active Ingredients** | **Inactive Ingredients** |
| --- | --- | --- | --- |
| **Group I** | Colgate kids anticavity toothpaste, Minions (Colgate, New York, USA) | Sodium Fluoride (0.132% w/v.) 600 ppm F | Sorbitol, water, hydrated silica, PEG-12, flavor, cellulose gum, tetrasodium pyrophosphate, sodium lauryl sulfate, sodium saccharin, CI 77019, CI 77891, CI 42090 |
| **Group II** | Darlie Jolly Junior (Hawley & Hazel Chemical Company, Hong Kong, Hong Kong SAR) | Sodium Monofluorophosphate (0.456%) 600 ppm F | Sorbitol, hydrated silica, flavor, PEG-12, sodium lauryl sulfate, tetrasodium pyrophosphate, carrageenan, benzyl alcohol, xylitol, sodium saccharin, dicalcium phosphate, titanium dioxide (CI 77891), CI 47005 |
| **Group III** | Elmex Kinder Zahnpasta (GABA International AG, Therwill, Switzerland) | Amine Fluoride 500 ppm F | Aqua, sorbitol, hydrated silica, hydroxyethylcullose, CI 77891, cocamidopropyl betaine, olaflur, aroma, saccharin, limonene |
| **Group IV** | Lion Kodomo (Lion, Tokyo, Japan) | 5% Xylitol and Sodium Fluoride 500 ppm F | -No mention- |
| **Group V** | Oral-B kids toothpaste (Procter & Gamble, Cincinnati, USA) | Sodium Fluoride 500 ppm F | Sorbitol, aqua, hydrated silica, sodium lauryl sulfate, trisodium phosphate, aroma, cellulose gum, sodium phosphate, sodium saccharin, carbomer, limonene, polysorbate 80, sodium hydroxide, CI 42090 |
